# Feature Selection on 2D and 3D Geometric Features to Improve Facial Expression Recognition

**DOI:** 10.3390/s20174847

**Published:** 2020-08-27

**Authors:** Vianney Perez-Gomez, Homero V. Rios-Figueroa, Ericka Janet Rechy-Ramirez, Efrén Mezura-Montes, Antonio Marin-Hernandez

**Affiliations:** Research Center in Artificial Intelligence, University of Veracruz, Sebastian Camacho No.5, Centro, Xalapa C.P. 91000, Mexico; vianneyapg@gmail.com (V.P.-G.); erechy@uv.mx (E.J.R.-R.); emezura@uv.mx (E.M.-M.); anmarin@uv.mx (A.M.-H.)

**Keywords:** facial expression recognition, feature selection, facial geometric features

## Abstract

An essential aspect in the interaction between people and computers is the recognition of facial expressions. A key issue in this process is to select relevant features to classify facial expressions accurately. This study examines the selection of optimal geometric features to classify six basic facial expressions: happiness, sadness, surprise, fear, anger, and disgust. Inspired by the Facial Action Coding System (FACS) and the Moving Picture Experts Group 4th standard (MPEG-4), an initial set of 89 features was proposed. These features are normalized distances and angles in 2D and 3D computed from 22 facial landmarks. To select a minimum set of features with the maximum classification accuracy, two selection methods and four classifiers were tested. The first selection method, principal component analysis (PCA), obtained 39 features. The second selection method, a genetic algorithm (GA), obtained 47 features. The experiments ran on the Bosphorus and UIVBFED data sets with 86.62% and 93.92% median accuracy, respectively. Our main finding is that the reduced feature set obtained by the GA is the smallest in comparison with other methods of comparable accuracy. This has implications in reducing the time of recognition.

## 1. Introduction

People use cognitive mechanisms to recognize emotions during the communication process. One cognitive mechanism is to understand the non-verbal human behavior. This has been investigated for decades since 1872 with the study of Darwin, which involves a cognitive analysis of facial expressions [1]. In 1978, Suwa et al. presented the first attempt to automatically analyze facial expressions [2]. In the same year, Ekman and Friesen proposed a standard called Facial Action Coding System (FACS). This standard is composed of 44 facial Action Units (AUs) describing all facial movements. Likewise, Ekman proposed the six basic universal emotions: happiness, sadness, surprise, fear, anger, and disgust [2,3].

Most facial emotional recognition (FER) approaches follow three main steps: (a) face and landmark detection, (b) feature extraction, and (c) classification [4,5,6].

The first step includes different tasks. Sometimes it can include preprocessing to reduce the noise, image enhancement, and normalization. However, the most important task is face detection [6]. Once this is done, features are located on the face. Common techniques in this step are masking, scaling, converting into grayscale, and the detection of landmarks and regions [4,7]. Considering the type of data, some works take an image only as input; therefore, they are static [8,9,10]. On the other hand, the dynamic type uses a temporal sequence of images [8,11].

Recently, robust landmark detection techniques have been developed that work in-the-wild conditions, on video sequences with variations in pose, illumination, and with occlusion conditions [12]. They use a combination of local and global motion modeling to locate and track features.

If the face is not in frontal view, some processing is required before feature extraction. As in the cases of large displacements (LD) and head pose variations (HPV) that happen in in-the-wild conditions, it is necessary to remove their effect to obtain invariance in the recognition. LDs introduce cinematic blur, scale changes, and translation, whereas HPVs are related to 3D rotations. The objective of face registration is to find the transformation which minimizes the differences between two or more faces. Usually a distorted face is registered into a frontal view face before feature extraction. The amount of LD and HPV as well as the face registration techniques have an impact on facial expression recognition [13].

The second step is feature extraction. The objective is to extract an effective and efficient representation [6], methods such as feature point, interpolation polynomials, local binary patterns, Gabor, Histogram of Oriented Gradients, and optical flow are used [4,7]. It is important to remark that this step can include feature selection or feature construction. Usually reduction methods are embedded in this section [7]. Many investigations have used different features and types of data. One category is based on appearance and employs texture of the skin and wrinkles. A second category uses a geometric approach that employs the localization and shape of facial components such as nose, eyes, eyebrows, and mouth [8]. Focusing on the geometric approach and considering the type of data it is possible to extract features in 2D [14,15] and 3D [11,16,17]. Using a dynamic approach with sequence of images and employing advanced local motion patterns, it has been possible to recognize micro- and macro-expression in-the-wild in a unified framework [18].

The last step is classification, in this step we can consider the training of the model and the classification of new instance. Different algorithms are used such as Neural Networks, C4.5, k-NN (k-Nearest Neighbors), AdaBoost, SVM, and more recently Deep Learning (DL), which has a different approach [4,7].

In the conventional FER approaches, features and classification methods are selected by the researcher. In contrast, deep learning (DL) approaches extract the features and classify them using an end-to-end learning framework. In this case, the researcher specifies the architecture and its parameters. Usually DL requires a lot of data to train and usually achieves a high degree of accuracy. When comparing both approaches, conventional ones require lower computational power, memory, and data than deep learning approaches. They are still being used for real-time embedded systems due to their low computational complexity and high degree of accuracy [4,19].

FER systems still face many challenges like recognizing spontaneous expressions in real-time and in unconstrained environments. This includes variations in skin color; changes in illumination; aging; head pose variation; occlusion of objects like scarf, hair, or glasses; and complex background [6].

**Figure 1:**
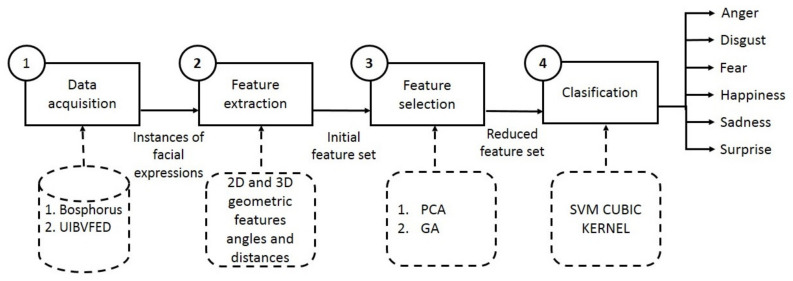
Methodology.

Our proposed cognitive system employs a traditional approach and follows four steps: data acquisition, feature extraction, feature selection, and classification (Figure 1). Considering the importance of the selection of features, we decide to separate the feature extraction and feature selection. This work is focused on the reduction of features to classify the six basic facial expressions: surprise, sadness, happiness, fear, disgust, and anger, while at the same time achieving a high degree of classification accuracy. Our study employs the static approach and uses instances of faces from the Bosphorus database in the step of feature extraction, and Bosphorus and UIVBFED databases, to train and test the model of classification.

The main contributions of our research are the following:

- A set of geometric features is proposed, evaluated, and compared. These features are derived from the 2D and 3D geometry of the human face using angles and normalized distances between landmark points of the face.
- To obtain relevant features PCA as common technique and a GA as a new proposal are implemented and compared.
- The performances of four classifiers (k-NN, E-KNN (ensemble classifier subspace K-nearest neighbor), SVM3 (SVM using a cubic kernel), and SVM2 (SVM using a quadratic kernel)) are compared employing our features.
- A comparative study is presented. Our proposal compares favorably in terms of accuracy with other works in the literature using static images and Bosphorus database and also greatly lowering the number of used features. Reducing the computational cost of the classification.

The rest of the paper is organized as follows. Section 2 describes material and methods, specifically, details regarding the data acquisition, feature extraction, feature selection, and classification. Section 3 presents our experiments and results. Finally, Section 4 and Section 5 show the discussion and the research findings.

## 2. Materials & Methods

Figure 1 presents our methodology, which consists of 4 stages: (i) data acquisition, (ii) feature extraction, (iii) feature selection, and (iv) classification.

In the data acquisition stage, instances of facial expressions are collected from the Bosphorus or UIBVFED database. Then, feature extraction is carried out and, from 3D landmarks on a human face, 89 geometric features are determined. In the third stage, relevant features are selected from the original feature set through two methods: PCA and a GA.

Finally, a support vector machine with cubic kernel is applied for classification to the original feature set and the reduced feature sets to classify the six basic facial expressions. The expression are (i) anger, (ii) disgust, (iii) fear, (iv) happiness, (v) sadness, and (vi) surprise. Through this paper, these facial expressions will be expressed as AN, DI, FE, HA, SA, and SU, respectively.

### 2.1. Data Acquisition

This step involves the process to get the facial information. In traditional FER approaches, the algorithms are trained and tested in a database. In unconstrained environments the efficacy depends on acquired image data and is limited for some issues such as occlusions, pose variation, and illumination [6].

For this study, we only consider the databases where a single face appears in each image and that 3D landmarks are identified. We extracted the information from two different datasets: (a) Bosphorus and (b) UIBVFED. Table 1 shows characteristics of these two databases [20,21].

#### 2.1.1. Bosphorus Facial Database

The Bosphorus database is commonly used in 3D facial expression recognition. This database was developed in 2008 by the University of Bogazici in Turkey [22]. The database includes a set of 4652 3D facial scans with manually defined landmarks collected from 105 subjects: 60 men and 45 women. The scans were taken under different occlusion conditions and various poses from the subjects. Furthermore, every subject was instructed to express the six basic emotions, the neutral state, and various AUs. Each scan covers just one pose or expression. These are acquired using a 3D sensor (InSpeck Mega Capturor II 3D) with a depth resolution of 0.3 mm (*X* axis), 0.3 mm (*Y* axis), and 0.4 mm (*Z* axis) [22,24].

For our experiments, 424 scans (instances) were taken. Specifically, Table 2 shows the number of instances obtained per facial expression (surprise, sadness, happiness, fear, disgust, and anger). The selected scans correspond to faces showing the six universal facial expressions. All these images are from frontal faces, with no pose variation, and without occlusions.

#### 2.1.2. Virtual Facial Expression Dataset UIBVFED

**Figure 2:**
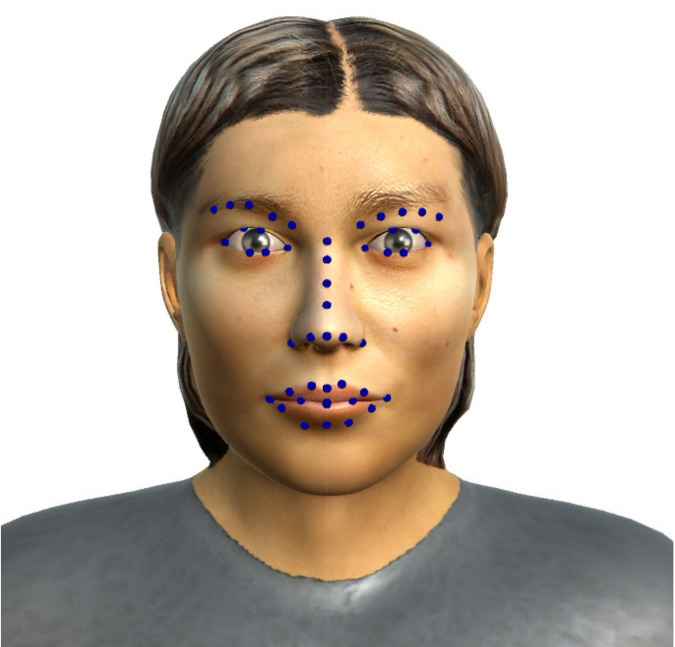
Landmarks in UIBVFED.

This data set Virtual Facial Expression Dataset UIBVFED was developed using Autodesk Character Generator and consist of 640 images and respective set of landmarks [23]. It corresponds to 51 points in the image in the 3D space (Figure 2).

In total, it contains 20 characters from different ethnicities, 10 men and 10 women, with ages between 20 and 80 years. Each avatar includes 32 expressions grouped in the six universal expressions [23].

**Figure 3:**
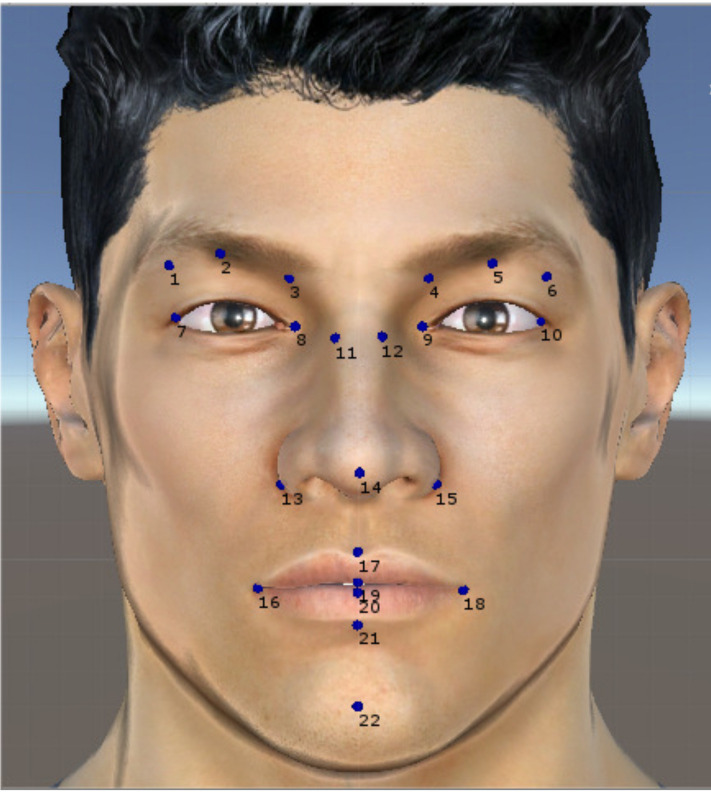
Twenty-two landmarks adapted in the UIBVFED database.

In our experiments we employed one expression from each of the six universal facial expressions and all the corresponding data from the avatars. In addition, to get the same landmarks from this database, we interpolated three landmarks and removed other landmarks that were not necessary, as illustrated in Figure 3.

### 2.2. Feature Extraction

Emotions are expressed through movements in certain regions of the face, which can be parameterized based on muscle movements. Up to now, two major facial parameterization systems have been developed. The first is the Facial Action Coding System (FACS) developed by Ekman and Friesen and the second is a component of the MPEG-4 standard, the Face Animation Parameters (FAP) [25].

FACS is an anatomical system to determine facial behaviors through changes in the muscles. These changes are denominated Action Units (AUs). The system contains 44 AUs; nevertheless, the six basic facial expressions can be represented using 18 AUs only [26,27].

**Figure 4:**
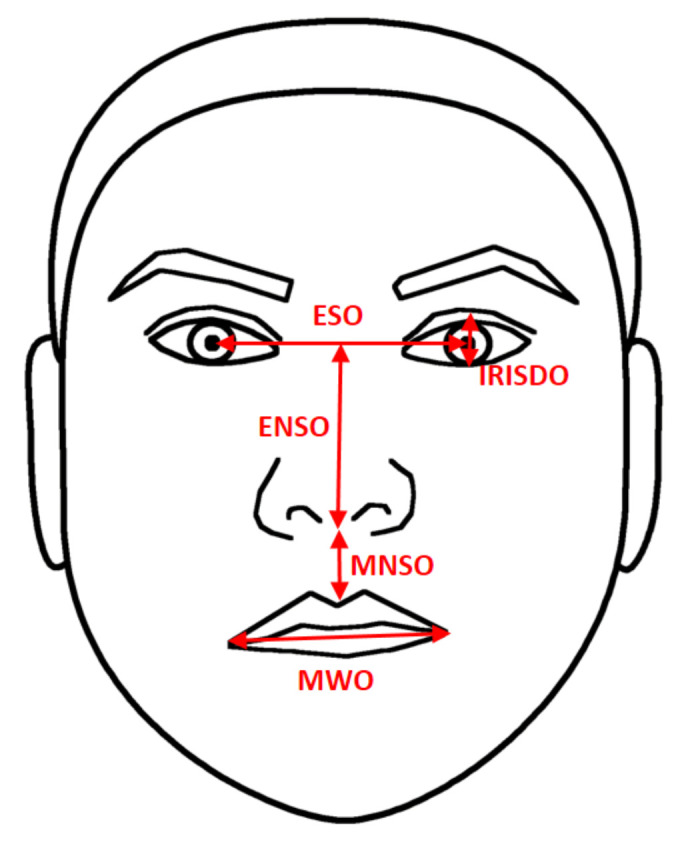
Measurements that define Facial Animation Parameter Units (FAPUs) based on the work in [28].

The second system is part of MPEG-4, this standard includes the Facial Definition Parameters (FDP) to specify the size and the shape of the face, and the Facial Animation Parameter (FAP), which is used to represent every facial expression. FAP is expressed in terms of Facial Animation Parameter Units (FAPUs). FAPUs are used to keep the proportionality. They are defined as distances between some key points in a neutral state [28,29]. According to the authors of [28], the measurements (Figure 4) are defined as follows.

- IRISDO: It is the distance between the upper and the lower eyelids, i.e., the approximate iris diameter.
- ESO: It is the distance between the eye pupils, i.e., eye separation.
- ENSO: It is the distance between the center of ESO and below the nostrils, i.e., eye–nose separation.
- MNSO: It is the distance between the upper lip and the nostrils, i.e., mouth–nose separation.
- MWO: It is the distance between the left corner and right corner of the lips, i.e., mouth width.

Furthermore, the MPEG-4 system specifies the movements related to the six basic facial expressions. Table 3 explains these basic facial expressions according to [29].

**Figure 5:**
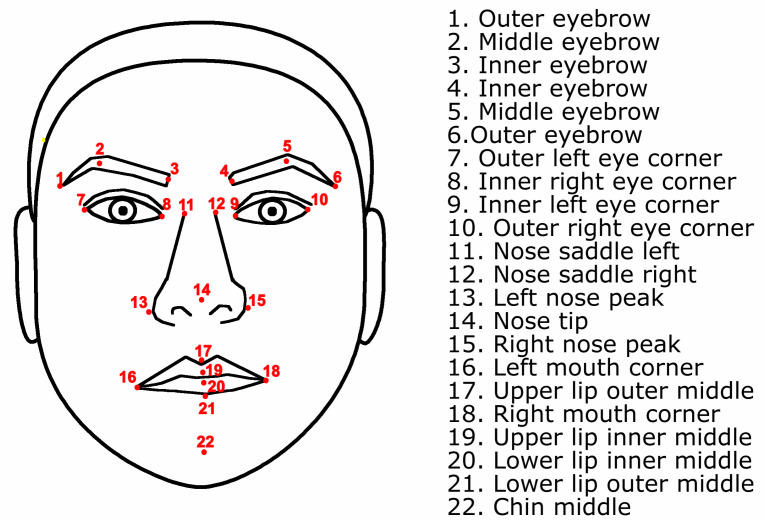
Twenty-two landmarks used in feature extraction.

Based on the two previous systems (FACS and MPEG-4), a set of features was defined by us using 22 of the 24 landmarks included in the Bosphorus database to represent the six basic facial expressions. Figure 5 shows these landmarks. In total, 89 features were defined: 27 angles and 19 distances in 3D, and 20 angles and 23 distances in 2D. Table 4 presents these 89 features with an identifier assigned to each one.

Euclidean distance (Equation (1)) was employed to compute distances between landmarks. Then, all distances were normalized using the distance between the landmarks 8 and 9, see Figure 4 and Figure 5.

(1) $$d\left(p_{1},p_{2}\right)=\left\|p_{1}-p_{2}\right\|$$

The angles were calculated applying cosine between two vectors with three points as shown in Equation (2).

(2) $$A=\arccos\left(\frac{\left(p_{3}-p_{2}\right)\cdot \left(p_{1}-p_{2}\right)}{\|p_{3}-p_{2}\left\|\right\|p_{1}-p_{2}\|}\right)$$

where $p_{i}$ are the X-Y-Z coordinates of the landmark for the 3D-features, and X-Y coordinates of the landmark for the 2D-features.

### 2.3. Feature Selection

An important component in data mining and machine leaning is feature selection. It is a difficult task because many features are used to obtain information; however, not all of them are essential and can reduce the performance of classification. The complexity of the problem deepens on the number of features, where the total number of possible solutions is $2^{n}$ for a dataset with *n* features. For this type of combinatorial optimization problem, evolutionary computation approaches are a good option [30].

Feature selection is a process where a subset of relevant features are selected from the original set, and it helps to choose the relevant features (removing irrelevant or redundant features) to understand the data, improve the predicted performance, and reduce computational requirements and the time of training [30,31].

A variety of techniques have been applied to feature selection and they are grouped in different ways as described below. Dimensionality reduction algorithms such as those based on projection (e.g., Fisher linear discriminant, principal component analysis, or compression (e.g., using information theory) modify the original representation of the features. In contrast, feature selection algorithms only select a subset of them. These algorithms can be classified into two groups, filter, and wrapper methods. The first class of methods uses general characteristics of the data to select a feature subset without using any learning algorithm. The second class manages a learning algorithm and uses its performance as the evaluation criterion. A new class of feature selection algorithms integrates the theory of sparse representation [31].

In this research, two methods are evaluated to select features: PCA employing a traditional implementation and a a GA which we designed. The GA operates as a wrapper feature selection method using a support vector machine.

#### 2.3.1. Principal Component Analysis (PCA) for Feature Selection

Principal Component Analysis (PCA) is a statistical technique widely used for dimensionality reduction. PCA converts the original set of variables into a smaller set of uncorrelated variables called principal components. These components provide the most representative information of the original set [32]. Algorithm 1 explains the steps performed in PCA to obtain the reduced feature set.
**Algorithm 1:** PCA algorithm  **Input** The set of original features  **Output** The new set of features1:Subtract the mean from each of the data dimensions2:Calculate the covariance matrix3:Calculate the eigenvectors and the eigenvalues4:Choose components with a percentage of variance to be used for creating a feature vector (in our experiments, 97%, 98%, and 99% were selected as the percentages of variance)5:Derivate the new data set

#### 2.3.2. Genetic Algorithm (GA)

Genetic Algorithms (GAs) are the first evolutionary computation techniques employed in feature selection. They have been applied in different applications which include image processing and in specific in face recognition [30].

GA are adaptive heuristic search algorithms and optimization methods based on evolutionary ideas of the natural selection, such as selection mechanism, recombination, and mutation [33]. In this research, a GA selected a subset of features.

**Figure 6:**
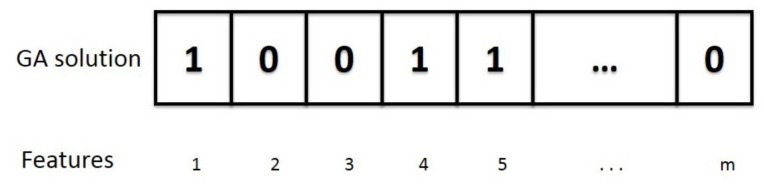
GA feature set solution representation.

In the algorithm, every individual in the population represents a possible subset of features. An individual is defined using a binary vector (0 = absence and 1 = presence) of m genes (Figure 6), where m is the number of features, in this case 89 features. The average accuracy using 10-fold cross-validation is the fitness function. The GA was implemented using the following parameters.

- Population size: 20
- Number of generations: 250
- Parent selection: the best two out of five randomly chosen individuals.
- Recombination: one point crossover
- Mutation: simple
- Use elitism to select the best individual

Algorithm 2 presents in detail the steps followed to reduce the original set via a GA.
**Algorithm 2:** Genetic algorithm  **Input** Population size, MAX GENERATION  **Output** The best individual in all generations1:INITIALIZE population with random candidate solution. The genes correspond to 2D and 3D features.2:EVALUATE each candidate solution where the fitness function is the percentage of correct classification of the SVM33:**while** the number or generation is less than MAX GENERATION **do**4:    Select the best two out of five randomly chosen individuals5:    Recombine pair of parents with one point crossover6:    Use simple mutation7:    Evaluate each candidate solution8:    Use elitism to select the best individual9:    Update the population for the next generation   

### 2.4. Classification

In our research, four classifiers were employed to distinguish the facial expressions: SVM3, SVM2, k-NN fine, and E-KNN. The simplest case of SVM classification is a binary learner which finds the optimal hyperplane that separates the points in the two classes. There are generalizations of SVM that use nonlinear functions to separate the two classes. In this case, it is common to use kernel functions such as a polynomial kernel: $K\left(x,y\right)={\left(1+x.y\right)}^{d}$, for some positive integer *d*, where *x* and *y* are feature vectors. Focusing on Matlab tool, a SVM2 uses d=2, whereas a SVM3 employs d=3. There are different approaches to build a multiclass classifier from binary classifiers, when C>2, where *C* stands for the number of different classes (multiclass classification problem). One such approach is the “one-versus-one” approach, where C(C−1)/2 different binary SVM classifiers are built for all possible pairs of classes. A new test point is classified according to the class that has the highest votes from all the binary classifiers [34]. This is the approach used in Matlab for multiclass SVM [35].

On the other hand, a k-Nearest Neighbor (k-NN) algorithm assigns a test pattern, *x*, to the most representative class of the *k*-nearest neighbors [36]. In Matlab, k-NN fine corresponds to a classification with k=1. Regarding the ensemble classifiers, these are methods that combine the output of multiple weak classifiers to produce a stronger classifier. One way to combine weak classifiers is using a weighted combination [36]. Moreover, the combination or ensemble method to be used depends on whether it is for classification of two classes or more classes. Matlab has the subspace method to combine the results of multiple weak classifiers. The subspace method works for two or more classes. The subspace method of combination can work with either discriminant analysis or k-NN as weak classifiers. In our experiments with classifiers, we have used ensemble classifiers with the subspace method for combination and using k-NN as weak classifiers. The subspace algorithm is a random algorithm that uses the following parameters; *m* is the number of dimensions to sample in each weak classifier, *d* is the number of dimensions or features in the data, and *n* is the number of weak classifiers in the ensemble. In our experiments, the default values used in Matlab are n=200, m=round(d/2). According to the authors of [35], the random subspace algorithm performs the following steps. “(1) Choose without replacement a random set of m predictors from the d possible values. (2) Train a weak learner using just the m chosen predictors. (3) Repeat steps 1 and 2 until there are n weak learners. (4) Predict by taking an average of the score prediction of the weak learners, and classify the category with the highest average score”.

## 3. Experiments and Results

For the experiments we will be using two databases: one is the Bosphorus database (Section 2.1.1), and the other is UIBVFED database (Section 2.1.2). The motivation for the experiments is to test the classification accuracy of the original feature set (see Section 2.2) and compare with two feature selected sets. One reduced feature set is obtained using PCA (see Section 2.3.1). The second reduced featured set is obtained using our proposed GA (see Section 2.3.2 ). The following experiments were done.

1. Assessment of the classification accuracy of the original feature set (see Section 2.2) using the Bosphorus database.
2. Selection of a reduced feature set using PCA and assessment of classification accuracy using the Bosphorus database.
3. Selection of a reduced feature set using our GA and assessment of classification accuracy using the Bosphorus database
4. Assessment of the classification accuracy of the reduced feature set using GA on the UIBVFED database.

In all experiments with the Bosphorus dataset the classes were balanced to 104 instances using the SMOTE algorithm.

### 3.1. Original Feature Set and Performance Evaluation

For the first experiment, 89 features were processed: 27 angles and 19 distances in 3D, and 20 angles and 23 distances in 2D. These features were extracted according to the experimental setting explained in Section 2.2.

To evaluate the accuracy and try to get the best classifier for our data, four classifiers (E-KNN, K-NN, and SVM using a cubic and a quadratic kernels) were employed. We test this experiments using 10-fold cross-validation technique. The Table 5 shows the results.

In general, all methods are above the 80% of accuracy, but it is important to note that the SVM3 reported the highest performance in the median, mean, maximum, and minimum. On the other hand, the lowest standard deviation was obtained by SVM2, but all classifiers are under 0.6.

Table 6 shows the percentage per emotion of the best classifier, SVM3. “Happiness” reported highest accuracy and “fear” the lowest accuracy.

### 3.2. Feature Selection Using PCA

From the previous experiments, it was noticed that SVM3 reported the highest accuracy in average. Therefore, experiments were performed using the SVM3 as the only classifier.

For these experiments, PCA was used to reduce the feature set. It can be seen from Table 7 that our feature set was reduced from 89 features to 21, 27, and 39 through PCA varying the percentage of variation of data dispersion. In this preliminary test, one execution of the SVM3 was performed per reduced feature set. It can be seen that PCA with 99% of variance obtained the highest accuracy (81.25%) employing 39 features.

In order to perform the best result of the reduction with PCA, we employed 10-fold cross-validation with SVM3. Table 8 shows the results.

The tabla shows Table 9 the confusion matrix. It can be noticed that “happiness” reports the best accuracy and “fear” the lowest accuracy followed by “disgust”.

### 3.3. Feature Selection Using GA

Similarly to the experiments with PCA, a GA was used to select a subset of features. The GA was executed 30 times to evaluate its performance using a SVM3 only. Table 10 shows the best and the worst fits from these 30 executions. It can be seen that the best fit achieved 89.58% accuracy on average using 47 features, whereas the worst fit obtained 87.82% accuracy on average using 46 features. Moreover, it is important to remark that in terms of number of features, the best fit only has one more feature than the worst fit. Nevertheless, it can be noticed that the best fit uses more 3D features than the worst fit, i.e., the best fit employs 26 3D features, whereas the worst fit uses 21 3D features.

**Figure 7:**
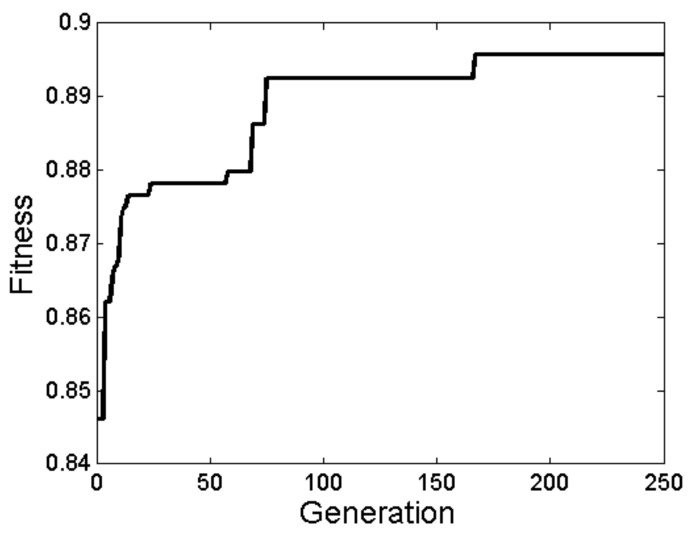
Convergence graph.

Focusing on the best fit, it can be seen from Figure 7 that after the 167th generation, the fitness function achieved 89.58% accuracy in average. Regarding the features obtained with the GA in the best fit, the reduced feature set is composed of 47 features: 14 angles and 12 distances in 3D and eight angles and 13 distances in 2D (Table 11).

Such as in the previous experiment we testes the featured set using 10-fold cross-validation and SVM3. The measures of the performance are reported in Table 12.

The confusion matrix (Table 13) shows the accuracy per emotion and we noticed that “happiness” has the best accuracy and “fear” the lowest accuracy.

### 3.4. Evaluation on UIBVFEd Dataset

To evaluate the performance of the reduced feature set obtained with the GA we trained and tested with UIBVFED database. For this experiment 10-fold cross-validation and SVM3 were employed.

The results obtained in this database (Table 14) were better than those obtained with Bosphorus database.

Regarding the accuracy per facial expression, Table 15 presents the confusion matrix. It can be noticed that “happiness”, “fear”, and “anger” expressions obtained perfect accuracy (100%), and sadness and surprise the lowest.

## 4. Discussion

### 4.1. Overall Performance of the Classifiers and Feature Sets

Table 16 shows the accuracy for every feature set. We can see that the best result is obtained with the GA using the UIBVFED dataset with the best percentage of accuracy and the lowest number of features followed by the experiment under the same conditions but with the Bosphorus database.

On the other hand, with the previous experiments and the confusion matrices, we noticed that with the Bosphorus database for all experiments the best performance per emotion is obtained for “happiness” and the second place is “sadness”. For Bosphorus database, in all experiments, the lowest accuracy was obtained by “fear”. In contrast, for the UIBVFED database “fear” achieved perfect accuracy.

### 4.2. Number of Features

Regarding the number of features in each feature set, it can be concluded that PCA reduced the original features by 57% (i.e., 89 features were decreased to 39). However, the mean of the accuracy values achieved through a SVM3 was decreased by 3.91 units (i.e., from 85.11% <original feature set> to 81.20% <reduced feature set with PCA>). On the other hand, the GA reduced the original features by 47.2% (i.e., 89 features were decreased to 47) and increased the mean of the accuracy values achieved through a SVM3 by 1.51 units (i.e., from 85.11% <original feature set> to 86.62% <reduced feature set with GA>).

Specifically comparing the original feature set with the reduced feature set obtained via GA, it can be seen from Table 17 that the highest reductions were in 2D (60%) and 3D angles (48.15%). Despite there was a reduction of 47.20% on the features.

An advantage to use less features is decrease in execution time. This can be seen in the last column of Table 17. The classification time of a new instance using the features obtained with the GA (47 features), is approximately 30% less than using the original feature set (89 features). To measure execution time we employed an Intel i7-4700MQ processor.

**Figure 8:**
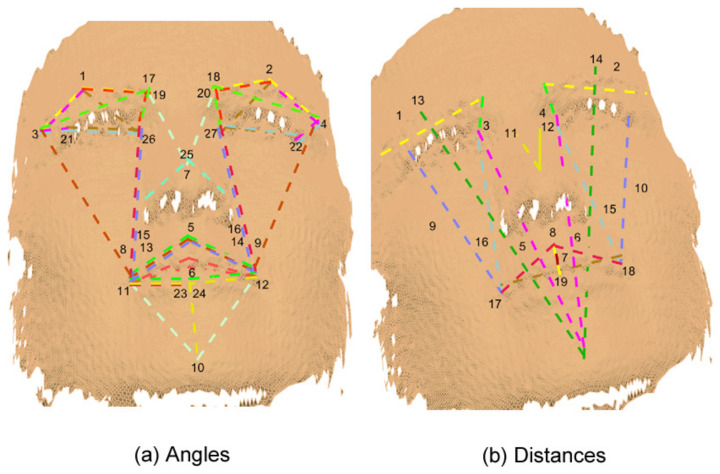
Original 3D features.

**Figure 9:**
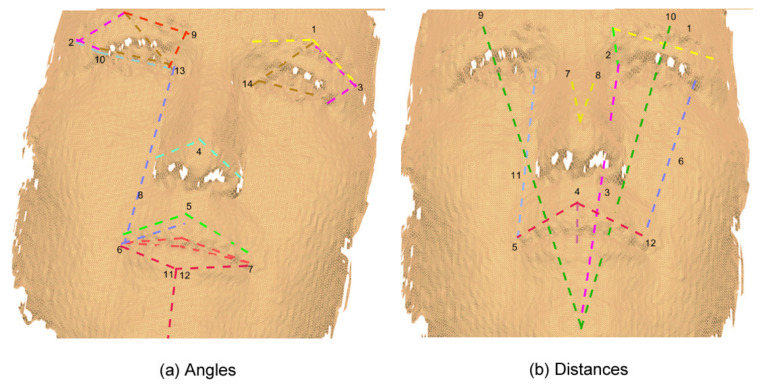
3D reduced features obtained through a GA.

**Figure 10:**
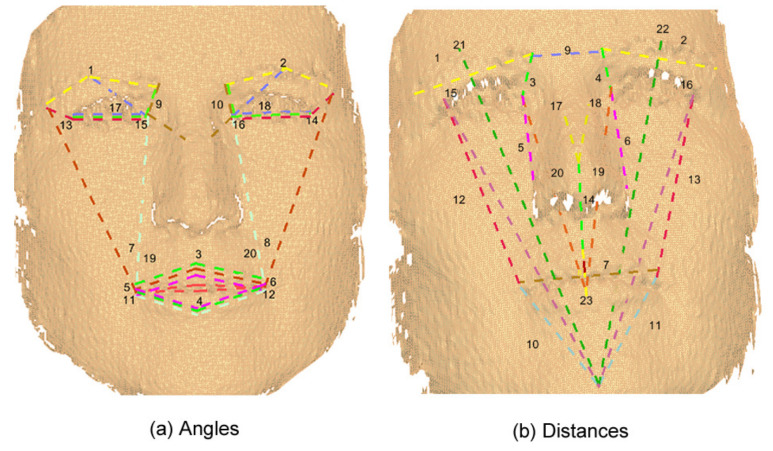
Original 2D features.

**Figure 11:**
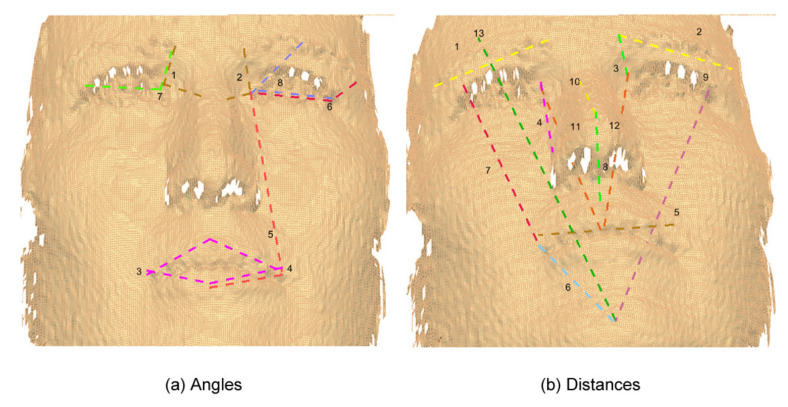
2D reduced features obtained through a GA.

Moreover, it can be noticed from Figure 8, Figure 9, Figure 10 and Figure 11 that the GA discarded features which have redundancy and only kept the features that are necessary for facial expression recognition, taking advantage that most of the human faces have symmetry.

### 4.3. Comparison of Our Results with Previous Studies

As explained earlier, this research used instances of faces from the Bosphorus database so that our results could be compared with previous studies.

#### 4.3.1. Comparison on Bosphorus Dataset with Handcrafted Features

The first three works were selected because they use 3D information and the same database to extract the features. Our highest median accuracy case obtained a better performance in average than Salahshoor and Faez [9] and Ujir [10]. Moreover, our proposal employs fewer features than the proposals of Salahshoor and Faez and Ujir. Conversely, our proposal, in its highest median accuracy case, is not as precise as Zhang et al. [11]. However, it is important to remark that the conditions are not the same: Zhang et al. used a dynamic approach. Compared to this last study, our proposal provides a better classification for happiness, fear, disgust, and sadness emotions. Moreover, our approach uses 47 features instead of 64 features employed in average by Zhang et al. (Table 18).

#### 4.3.2. Comparison on Bosphorus Dataset with Deep Features

As mention before, our work is focused in the selection of features. For this reason, we also compare with DL approaches, where the algorithms learn the features. We only compare with DL works which use Bosphorus dataset. The authors of [37,38] use the same database and type of data. Table 19 shows the comparison. In terms of accuracy, our method gets better performance, and the meaning of the features is clear.

#### 4.3.3. Comparison with Handcrafted Feature Methods on Other Datasets

The other datasets used in this study (Table 20) were chosen because they are focused in selecting relevant features. For example, Gaulart et al. [39] used a PCA for dimensionality reduction and Fast Neighbor Component Analysis (FNCA) for feature selection; in our case, GA made both tasks. In terms of accuracy on average compared with the above study, we achieved a greater accuracy of 93.92% versus 89.98%, and just 4.55% less than the best accuracy reported by Oh and Kim [40]. However, our method used less features. This is an advantage not just to train the model (lower time and computational cost). It is an important aspect in real-time applications.

## 5. Conclusions and Future Work

In this paper, we proposed a cognitive system for emotion recognition, where the main difference is observed in the selection of features. From these we get a novel and reduced set of geometric features in 2D and 3D to classify the six basic facial expressions: happiness, sadness, surprise, fear, anger, and disgust. The original feature set was reduced using PCA and GA. Furthermore four classifiers (cubic and quadratic SVM, k-NN, and E-KNN) were applied to the original feature set. The best performance was obtained through a GA using a SVM3 with 10-fold cross-validation, which reduced the number of features by 47.20%, i.e., from 89 to 47 features, and obtained 93.92% and 95.83% as the highest mean accuracy and the highest maximum accuracy, when tested on the UIBVFED dataset. It is important to remark that the GA was able to discard features having redundancy and could detect relevant features based on the symmetry presented on most of the human faces. Finally, the key contribution of this research is that emotions might be recognized with 93.92% accuracy using only 47 features. This proposal might be employed to improve the computational cognitive mechanism used to infer emotions in a static image; therefore, a computer might adapt its interaction with people based on the emotion detected. As a future work, we will investigate the value of the proposed descriptors on more datasets and possibly explore micro-expressions.

## Figures and Tables

**Table 1:** 3D facial expressions datasets.

Database	Samples	Subject	Content	Temporality
Bosphorus [22]	4652	105	Poses with different occlusion conditions	Static
			and the six basic expressions and the neutral state	
UIBVFED: Virtual facial	640	20	32 expressions	Static
expression dataset [23]				

**Table 2:** Number of instances per facial expression.

Facial Expression	Instances
Surprise	63
Sadness	66
Happiness	99
Fear	62
Disgust	64
Anger	70
Total	424

**Table 3:** Description of the six basic facial expressions.

Facial Expression	Eyebrows	Inner Eyebrows	Eyes	Mouth	Jaw
Anger	-	Pulled downward and together	Wide open	Lips are pressed against each other or opened to expose the teeth	-
Disgust	Relaxed	-	Eyelids: relaxed	Upper lip: raised and curled, frequently asymmetric	-
Fear	Raised and pulled together	Bent upward	Tense and alert	-
Happiness	Relaxed	-	-	Open. Corners of the mouth: pulled back toward the ears	-
Sadness	-	Bent upward	Slightly closed	Relaxed	-
Surprise	Raised	-	Upper eyelids: wide open Lower eyelids: relaxed	-	Open

**Table 4:** 3D and 2D original features.

3D Features					2D Features				
**#**	**ID**	**Dist**	**ID**	α	**#**	**ID**	**Dist**	**ID**	α
1	1D3	$p_{1},p_{3}$	1A3	$p_{1},p_{2},p_{3}$	1	1D2	$p_{1},p_{3}$	1A2	$p_{1},p_{2},p_{3}$
2	2D3	$p_{4},p_{6}$	2A3	$p_{4},p_{5},p_{6}$	2	2D2	$p_{4},p_{6}$	2A2	$p_{4},p_{5},p_{6}$
3	3D3	$p_{3},p_{8}$	3A3	$p_{2},p_{1},p_{7}$	3	3D2	$p_{3},p_{8}$	3A2	$p_{16},p_{17},p_{18}$
4	4D3	$p_{4},p_{9}$	4A3	$p_{10},p_{6},p_{5}$	4	4D2	$p_{4},p_{9}$	4A2	$p_{16},p_{21},p_{18}$
5	5D3	$p_{8},p_{22}$	5A3	$p_{16},p_{17},p_{18}$	5	5D2	$p_{8},p_{13}$	5A2	$p_{17},p_{16},p_{21}$
6	6D3	$p_{9},p_{22}$	6A3	$p_{16},p_{21},p_{18}$	6	6D2	$p_{9},p_{15}$	6A2	$p_{17},p_{18},p_{21}$
7	7D3	$p_{18},p_{16}$	7A3	$p_{13},p_{14},p_{15}$	7	7D2	$p_{18},p_{16}$	7A2	$p_{17},p_{16},p_{1}$
8	8D3	$p_{17},p_{21}$	8A3	$p_{17},p_{16},p_{1}$	8	8D2	$p_{17},p_{21}$	8A2	$p_{17},p_{18},p_{6}$
9	9D3	$p_{16},p_{7}$	9A3	$p_{17},p_{18},p_{6}$	9	9D2	$p_{3},p_{4}$	9A2	$p_{3},p_{8},p_{11}$
10	10D3	$p_{10},p_{18}$	10A3	$p_{16},p_{22},p_{18}$	10	10D2	$p_{16},p_{22}$	10A2	$p_{4},p_{9},p_{12}$
11	11D3	$p_{14},p_{11}$	11A3	$p_{19},p_{16},p_{20}$	11	11D2	$p_{18},p_{22}$	11A2	$p_{19},p_{16},p_{20}$
12	12D3	$p_{14},p_{12}$	12A3	$p_{19},p_{18},p_{20}$	12	12D2	$p_{7},p_{16}$	12A2	$p_{19},p_{18},p_{20}$
13	13D3	$p_{22},p_{2}$	13A3	$p_{8},p_{16},p_{21}$	13	13D2	$p_{10},p_{18}$	13A2	$p_{1},p_{7},p_{8}$
14	14D3	$p_{22},p_{5}$	14A3	$p_{9},p_{18},p_{21}$	14	14D2	$p_{17},p_{14}$	14A2	$p_{6},p_{10},p_{9}$
15	15D3	$p_{9},p_{18}$	15A3	$p_{8},p_{16},p_{17}$	15	15D2	$p_{7},p_{22}$	15A2	$p_{7},p_{8},p_{3}$
16	16D3	$p_{8},p_{16}$	16A3	$p_{9},p_{18},p_{17}$	16	16D2	$p_{10},p_{22}$	16A2	$p_{4},p_{9},p_{10}$
17	17D3	$p_{17},p_{16}$	17A3	$p_{1},p_{3},p_{8}$	17	17D2	$p_{14},p_{11}$	17A2	$p_{7},p_{8},p_{2}$
18	18D3	$p_{17},p_{18}$	18A3	$p_{9},p_{4},p_{6}$	18	18D2	$p_{14},p_{12}$	18A2	$p_{10},p_{9},p_{5}$
19	19D3	$p_{19},p_{20}$	19A3	$p_{2},p_{3},p_{8}$	19	19D2	$p_{9},p_{21}$	19A2	$p_{8},p_{16},p_{21}$
20			20A3	$p_{5},p_{4},p_{9}$	20	20D2	$p_{8},p_{21}$	20A2	$p_{9},p_{18},p_{21}$
21			21A3	$p_{1},p_{7},p_{8}$	21	21D2	$p_{22},p_{2}$	
22			22A3	$p_{6},p_{10},p_{9}$	22	22D2	$p_{22},p_{5}$	
23			23A3	$p_{16},p_{21},p_{22}$	23	23D2	$p_{19},p_{20}$	
24			24A3	$p_{18},p_{21},p_{22}$	24			
25			25A3	$p_{3},p_{14},p_{4}$	25			
26			26A3	$p_{7},p_{8},p_{2}$	26			
27			27A3	$p_{10},p_{9},p_{5}$	27			

**Table 5:** Accuracy of classification (original feature set).

Measure	Classifier			
	SVM3	SVM2	kNN	E-KNN
Standard deviation	0.50	0.38	0.55	0.48
Median accuracy	85.25	83.17	83.01	84.61
Mean accuracy	85.11	83.17	83.07	84.65
Maximum accuracy	85.73	83.81	84.29	85.41
Minimum accuracy	84.29	82.53	82.21	83.81

**Table 6:** Confusion matrix: 89 features.

%	SU	SA	HA	FE	DI	AN
**SU**	**79**	0	1	18	2	0
**SA**	0	**90**	0	1	3	6
**HA**	0	0	**97**	1	2	0
**FE**	12	3	0	**78**	7	1
**DI**	0	5	1	4	**84**	7
**AN**	0	12	0	1	4	**84**

**Table 7:** Feature selection using principal component analysis (PCA).

Reduced Features Using PCA			
% Variance	97%	98%	99%
Accuracy	75.48%	77.24%	81.25%
Features	21	27	39

**Table 8:** SVM3 (reduced feature set using PCA).

Measure	
Standard deviation	1.03
Median accuracy	81.08
Mean accuracy	81.20
Maximum accuracy	82.85
Minimum accuracy	79.16

**Table 9:** Confusion matrix: 39 features.

%	SU	SA	HA	FE	DI	AN
**SU**	**81**	0	0	15	4	0
**SA**	0	**83**	0	1	9	8
**HA**	0	0	**91**	1	8	0
**FE**	15	4	0	**75**	5	1
**DI**	2	11	2	4	**76**	6
**AN**	0	12	0	2	5	**82**

**Table 10:** Description of features and accuracy for the best and the worst fits.

	3D Features			2D Features			Number of Features	Average Accuracy
	α	Dist	Total	α	Dist	Total		
Worst fit	12	9	21	12	13	25	46	87.82%
Best fit	14	12	26	8	13	21	47	89.58%

**Table 11:** 3D and 2D features obtained through a genetic algorithm (GA).

3D Features					2D Features				
**#**	**ID**	**Dist**	**ID**	α	**#**	**ID**	**Dist**	**ID**	α
1	2D3	$p_{4},p_{6}$	2A3	$p_{4},p_{5},p_{6}$	1	1D2	$p_{1},p_{3}$	5A2	$p_{17},p_{16},p_{21}$
2	4D3	$p_{4},p_{9}$	3A3	$p_{2},p_{1},p_{7}$	2	2D2	$p_{4},p_{6}$	9A2	$p_{3},p_{8},p_{11}$
3	6D3	$p_{9},p_{22}$	4A3	$p_{10},p_{6},p_{5}$	3	4D2	$p_{4},p_{9}$	10A2	$p_{4},p_{9},p_{12}$
4	8D3	$p_{17},p_{21}$	5A3	$p_{16},p_{17},p_{18}$	4	5D2	$p_{8},p_{13}$	12A2	$p_{19},p_{18},p_{20}$
5	9D3	$p_{16},p_{7}$	7A3	$p_{13},p_{14},p_{15}$	5	7D2	$p_{18},p_{16}$	14A2	$p_{6},p_{10},p_{9}$
6	10D3	$p_{10},p_{18}$	11A3	$p_{19},p_{16},p_{20}$	6	10D2	$p_{16},p_{22}$	15A2	$p_{7},p_{8},p_{3}$
7	11D3	$p_{14},p_{11}$	12A3	$p_{19},p_{18},p_{20}$	7	12D2	$p_{16},p_{7}$	18A2	$p_{10},p_{9},p_{5}$
8	12D3	$p_{14},p_{12}$	15A3	$p_{8},p_{16},p_{17}$	8	14D2	$p_{17},p_{14}$	20A2	$p_{9},p_{18},p_{21}$
9	13D3	$p_{22},p_{2}$	19A3	$p_{2},p_{3},p_{8}$	9	16D2	$p_{10},p_{22}$		
10	14D3	$p_{22},p_{5}$	21A3	$p_{1},p_{7},p_{8}$	10	17D2	$p_{14},p_{11}$		
11	16D3	$p_{8},p_{16}$	23A3	$p_{16},p_{21},p_{22}$	11	19D2	$p_{9},p_{21}$		
12	18D3	$p_{17},p_{18}$	24A3	$p_{18},p_{21},p_{22}$	12	20D2	$p_{8},p_{21}$		
13			26A3	$p_{7},p_{8},p_{2}$	13	21D2	$p_{2},p_{22}$		
14			27A3	$p_{10},p_{9},p_{5}$	14				

**Table 12:** SVM3 (reduced feature set using GA).

Measure	
Standard deviation	0.73
Median accuracy	86.69
Mean accuracy	86.62
Maximum accuracy	87.17
Minimum accuracy	85.25

**Table 13:** Confusion matrix: 47 features.

%	SU	SA	HA	FE	DI	AN
**SU**	**81**	0	0	16	3	0
**SA**	0	**93**	0	1	5	1
**HA**	0	0	**96**	1	3	0
**FE**	16	1	0	**77**	6	0
**DI**	0	4	0	2	**89**	5
**AN**	0	11	0	0	6	**84**

**Table 14:** SVM3 (GA and database UIBVFED).

Measure	
Standard deviation	1.11
Median accuracy	93.75
Mean accuracy	93.92
Maximum accuracy	95.83
Minimum accuracy	92.50

**Table 15:** Confusion matrix: 47 features employed UIBVFED database.

%	SU	SA	HA	FE	DI	AN
**SU**	**85**	0	0	15	0	0
**SA**	0	**85**	0	0	15	0
**HA**	0	0	**100**	0	0	0
**FE**	0	0	0	**100**	0	0
**DI**	0	5	0	0	**95**	0
**AN**	0	0	0	0	0	**100**

**Table 16:** Mean of classification.

	Acuraccy	Features	Database
Original feature set	85.11%	89	Bosphorus
Best PCA	81.2%	39	Bosphorus
GA	86.62%	47	Bosphorus
GA	93.92%	47	UIBVFED

**Table 17:** Comparison between the original and reduced feature sets.

	3D Features		2D Features			Time to Classify
	α	Dist	α	Dist	Total	New Instance (ms)
Original feature set	27	19	20	23	89	0.00046364
Reduced feature set obtained via a GA	14	12	8	13	47	0.00032031
Reduction percentage	48.15%	36.84%	60%	43.48%	47.2%	

**Table 18:** Comparison on Bosphorus Dataset with handcrafted features.

	Proposed GA	Salahshoor & Faez (2012)	Ujir (2013)	Zhang et al. (2015)
Approach	Static	Static	Static	Dynamic
Classes	6	6	6	6
Feature Selection Methods	GA	None	mRMR	mRMR
Features	47	21600	115	64
Decision Methods	SVM	Modified Knn	Voting Schema (SVM’s)	Adaptive Ensemble Classifier
Accuracy (%)	86.62%	85.36%	66%	92.2%

**Table 19:** Comparison on Bosphorus Dataset with deep features.

	Proposed GA	Li et al. (2017)	Kun Tin (2019)
Data	2D + 3D	2D + 3D	2D + 3D
Features	47	32-D Deep Feature	Deep Feature Fusion
Classifier	SVM3	SVM	CNN
Accuracy	86.62%	79.17%	80.28%

**Table 20:** Comparison with other handcrafted feature method on different datasets.

	Proposed Median Accuracy	Proposed Median Accuracy	Goulart et al. (2019)	Oh & Kim (2020)
Approach	Static	Static	Static	Dynamic
Data Base	Bosphorus	UIBVFED	Cohn–Kanade + Preprocesing database	Own
Classes	6	6	7	5
Feature Selection Methods	GA	GA	PCA + FNCA	Grid Map
Features	47	47	60	2912
Decision Methods	SVM	SVM	SVM	ECOC-SVM
Accuracy (%)	86.62%	93.92%	89.98%	98.47%

## Abbreviations

The following abbreviations are used in this manuscript.

FACS	Facial Action Coding System
FER	Facial Emotional Recognition
LD	Large Displacements
HPV	Head Pose Variations
DL	Deep Learning
AUs	Action Units
SVM	Support Vector Machine
k-NN	k-Nearest Neighbors
AN	Anger
DI	Disgust
FE	Fear
HA	Happiness
SA	Sadness
SU	Surprise
FAP	Face Animation Parameters
FDP	Facial Definition Parameters
FAPUs	Facial Animation Parameter Units
FA	Factor Analysis
PCA	Principal Component Analysis
FLD	Fisher Linear Discriminant
GA	Genetic Algorithm
E-KNN	Ensemble classifier with subspace using k-NN
SVM3	Cubic Support Vector Machines
SVM2	Quadratic Support Vector Machines
α	angles
Dist	distance
